# Influence of the Growing Region on the Phytochemical Composition and Antioxidant Properties of North American Cranberry Fruit (*Vaccinium macrocarpon* Aiton)

**DOI:** 10.3390/plants12203595

**Published:** 2023-10-17

**Authors:** Liang Xue, Maureen Otieno, Kimberly Colson, Catherine Neto

**Affiliations:** 1 Department of Chemistry and Biochemistry, University of Massachusetts Dartmouth, North Dartmouth, MA 02747, USA; lxue1@umassd.edu (L.X.); motieno1@umassd.edu (M.O.); 2 Bruker BioSpin Corp., Billerica, MA 01821, USA; kimcolson.sebago@gmail.com

**Keywords:** cranberry, *Vaccinium*, flavonoids, antioxidant, triterpenoids, anti-inflammatory, growing region, climate

## Abstract

The impact of the growth environment on the production of health-promoting phytochemicals in cranberry fruit (*Vaccinium macrocarpon* Aiton) is not well established despite increased production worldwide. We investigated the secondary metabolite composition among the cranberry fruit of nine cultivars produced in two major coastal North American growing regions that differ in climate. Using ^1^H NOESY NMR to generate metabolic fingerprints, principal component analysis revealed variation between the two regions and identified likely contributing metabolites. Triterpenoids ursolic and oleanolic acid, as well as citric and malic acids, were quantified using ^1^H qNMR, and anthocyanins and flavonols were determined by HPLC-DAD. Total proanthocyanidins (PACs), total soluble phenolics, and DPPH free-radical scavenging antioxidant activity were also evaluated. Across all cultivars, anthocyanins, flavonols, and total phenolic content were significantly higher in West Coast fruit than East Coast fruit, correlating with a regional trend of higher antioxidant activity in fruit grown on the West Coast. The opposite trend was observed for triterpenoids and organic acids, which were significantly higher across cultivars in East Coast fruit. These trends persisted over two growing seasons. The study demonstrates that climate plays an important role in the production of antioxidant and anti-inflammatory phytochemicals in cranberry plants.

## 1. Introduction

Cranberry (*Vaccinium macrocarpon*) is a native fruit of North America belonging to the Ericaceae family, with increasing worldwide production and consumption due to its unique flavor and potential health benefits. The United States ranks as the top producer worldwide of cranberries (*Vaccinium macrocarpon*), followed by Canada, Chile, Turkiye, and Azerbaijan [1], and within the U.S., the top four producers by state are Wisconsin, Massachusetts, New Jersey, and Oregon [2]. Potential health benefits associated with cranberry consumption include promotion of urinary, oral, and gut health, as well as antioxidant and anti-inflammatory properties [3]. Clinical research studies indicate that consumption of cranberry, like other polyphenol-rich fruit, can positively impact serum lipid levels, blood pressure, oxidative damage to neurons and the vascular endothelium, and reduce the risk of cardiovascular disease [4,5]. Preclinical studies also suggest that cranberry has the potential to limit some cancers, including esophageal and inflammation-induced colon cancers [6,7,8,9].

Cranberry bioactivities are linked to the presence of multiple phytonutrients in cranberry fruits, notably flavonoids and triterpenoids. Anthocyanins, flavonols, and proanthocyanidins (PACs) are the major flavonoids found in cranberries [10]. Anthocyanins from various berries, including cranberries, were reported to possess powerful antioxidant and anti-inflammatory properties, for example, reducing inflammatory and oxidative stresses in BV-2 microglia [11]. Quercetin and its glycosides, abundant flavonols in cranberry fruit, are known for their antioxidant and anti-inflammatory properties and are likely to contribute to their health benefits [10]. Cranberry’s most abundant polyphenols are A-type PACs, of considerable interest due to their ability to interfere with adhesion and colonization of the bladder and urinary tract by extraintestinal pathogenic *Escherichia coli* and promote urinary and gut health [3]. We have recently demonstrated the ability of both polyphenol-rich and non-polyphenol-rich cranberry extracts to reduce inflammation and tumorigenesis in a mouse model of colitis [12]. Non-polyphenols of interest from a health perspective include ursolic acid and its isomer, oleanolic acid, two major triterpenoids present in the outer layer of the cranberry fruit that have recognized anti-inflammatory properties [13,14], and other health benefits related to mitigating oxidative stress [15]. Among *Vaccinium* fruit, cranberries are a rich source of ursolic acid and its esters, for which in vitro anti-tumor activities were previously reported [16]. These likely contribute to cranberries’ observed colon-protective properties.

Over 100 cultivars of cranberries are grown in North America [17], with a wide variety cultivated in Wisconsin and on the Eastern and Western coasts of the United States, primarily in Massachusetts, New Jersey, and Oregon. Massachusetts, the second largest producer of cranberries, grew approximately 23% of the cranberry crop in 2016 and 2017; Oregon contributed about 4–6% [18]. Phytochemical content in plants can be expected to vary due to genetic factors. Several previously published studies have compared the phytochemical profiles of different cranberry cultivars, but little data exists on regional influences. Previous studies have demonstrated that North American cranberry cultivars can vary significantly in the content of PACs, anthocyanins, flavonols, and organic acids [19,20,21]. For example, Canadian researchers characterized the phytochemical content diversity among five cranberry fruit cultivars grown in British Columbia, particularly anthocyanins, using HPLC and UPLC-MS-TOF profiling with chemometrics [19]. Three newer cranberry hybrids developed at Rutgers, Crimson Queen (Stevens × Ben Lear), Demoranville (Franklin (Early Black × Howes) × Ben Lear), and Mullica Queen (No. 35 (Howes × Searles) × Lemunyon), and other cultivars were examined for flavonoid, PAC, and organic acid content [20]. Triterpenoid content may also vary, but limited data is available [16]. In addition to genetic differences between fruit cultivars, variation in seasonal growing conditions between regions is also expected to affect the content of secondary metabolites in the fruit, since differences in climate may impact metabolic pathways in the plant. However, few studies have examined how regional factors impact cranberry phytochemical content. A recent study of eight different berries harvested in both Romania and Russia found significant differences in polyphenol content between samples from the two regions, which impacted their antioxidant and antibacterial properties [22].

The goal of this study was to determine how secondary metabolite composition varies between cranberry fruit produced in two major North American growing regions. Massachusetts on the East Coast and Oregon on the West Coast are approximately 3000 miles (4800 km) apart and vary substantially in climate, including rainfall, temperature, and humidity, providing an opportunity to study the resulting differences in fruit phytochemistry of common cultivars. To characterize variations in metabolite composition between regions, ^1^H NOESY NMR spectra of extracts were acquired, and principle component analysis (PCA) was used to identify key metabolites contributing to variation between sample sets. These metabolites were quantitated using qNMR and other established methods, and regional data were compared. The antioxidant activities of the extracts were evaluated using the DPPH free-radical scavenging assay and correlated with metabolite content. The data illustrates regional influences on phytochemicals in cranberry fruit associated with potential health benefits.

## 2. Results and Discussion

### 2.1. Non-Targeted PCA of Cranberry ^1^H NMR Reveals Regional Differences

**Figure 1:**
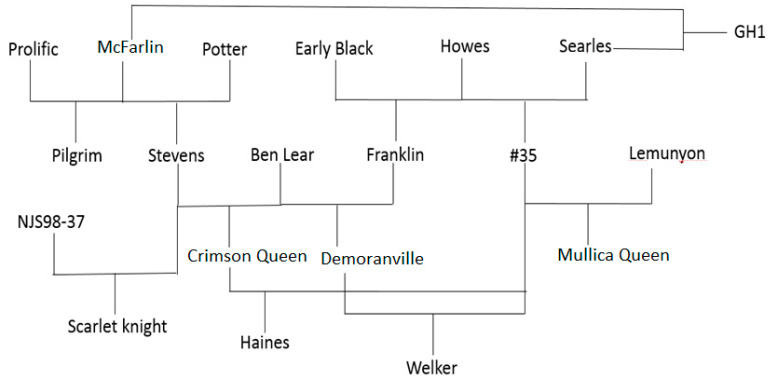
Breeding map showing the origins of Vaccinium macrocarpon cultivars included in the study and other common cultivars.

The present study examined variations in both polyphenol and non-polyphenol constituents of cranberry between multiple cultivars in two major growing regions. The genetic origins of the cultivars are shown in Figure 1. We employed ^1^H NMR in a non-targeted approach to reveal some differences between sample sets, then followed up with quantification of key metabolites using qNMR and other quantitative methods. Non-polyphenol metabolites of interest in cranberries include triterpenoids, due to their anti-inflammatory properties and potential impacts on carcinogenesis [16,23]. For PCA, samples were grouped in sets either by region or by cultivar.

NMR can provide a profile of all metabolites present in plant extracts in a single experiment without requiring chromatographic separations. Chemometric analysis of ^1^H NMR spectra has been used successfully to detect metabolic variations among plant sources, for example, ginsenoside and sucrose content across Ontario ginseng landraces [24], and for fingerprinting of Vaccinium species [25]. We recently reported the application of ^1^H NMR to compare commercial cranberry supplements to authentic cranberry standards and to identify constituents contributing to sample variation [26]. Principal component analysis considers all included data points within the spectrum and determines individual sources of variation (principal components, or PCs), which contribute to overall differences between populations. ^1^H NOESY NMR data were collected for all fruit extracts (n = 5 per treatment) and analyzed using Bruker AssureNMR^TM^ 2.0 and AMIX^TM^ 3.9.15, using methods we developed previously [26].

**Figure 2:**
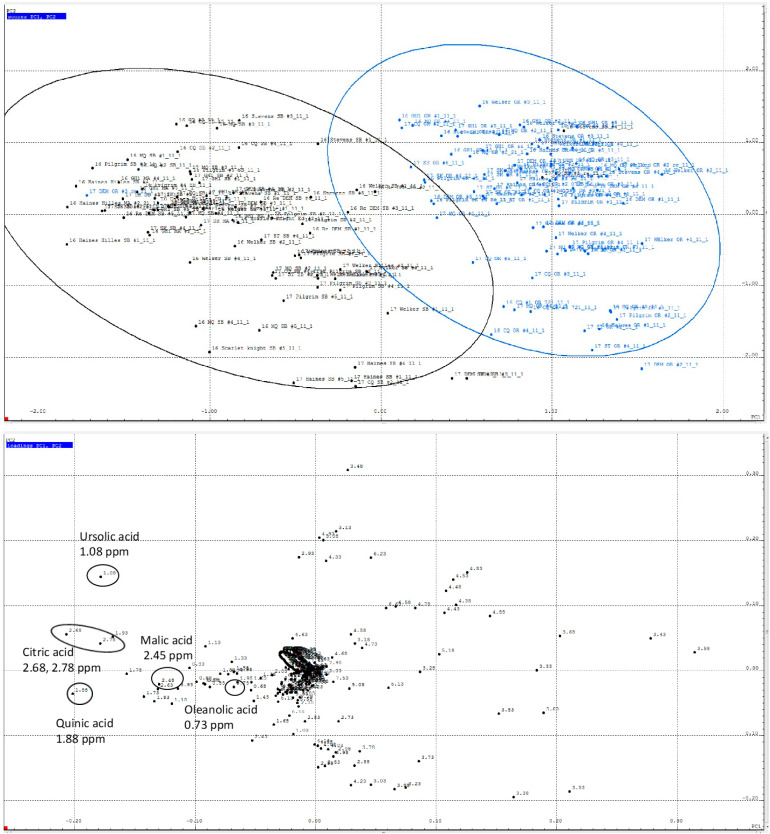
Principal component analysis (PCA) of ^1^H NOESY NMR spectra in the chemical shift range of 0.6–12.5 ppm. (**Top**) Scores plot showing a comparison of all samples by growing region (PC1 vs. PC2). Black dots represent MA samples; blue dots represent OR samples. (**Bottom**) Loadings plot showing the contributions of each chemical shift bucket (black dots) to the regional variance and identification of selected metabolites. Chemical shift buckets corresponding to the labeled metabolites are circled.

PCA of all cranberry fruit samples (Figure 2) revealed variations associated with the two different growing regions, for which PC1 accounts for 25.88% and PC2 for 21.44% of the sample variance. The separation of the two groups is evident in the score plot. Cranberry metabolites contributing to these differences were identified by comparing chemical shift buckets on the loadings plot with data in the SBASE. Buckets appearing prominently in the loadings plot and contributing significantly to variations between MA and OR cranberry fruit include chemical shifts associated with ursolic acid, oleanolic acid, malic acid, and citric acid. Their contributions were subsequently confirmed by quantification of the analytes using ^1^H NMR, discussed below. Chemical shifts between 3.3 and 3.8 ppm are associated with sugars, which also appear to play a role in the variations between the two regions; however, the individual sugars were not identified. A 2018 study of ten cranberry cultivars harvested in New Jersey used nontargeted metabolomic analysis of UPLC-HRMS data to characterize variations in phenolics among the cultivars [27]. In the present study, when data on samples from both regions were combined, no significant separation of samples by cultivar was observed.

**Figure 3:**
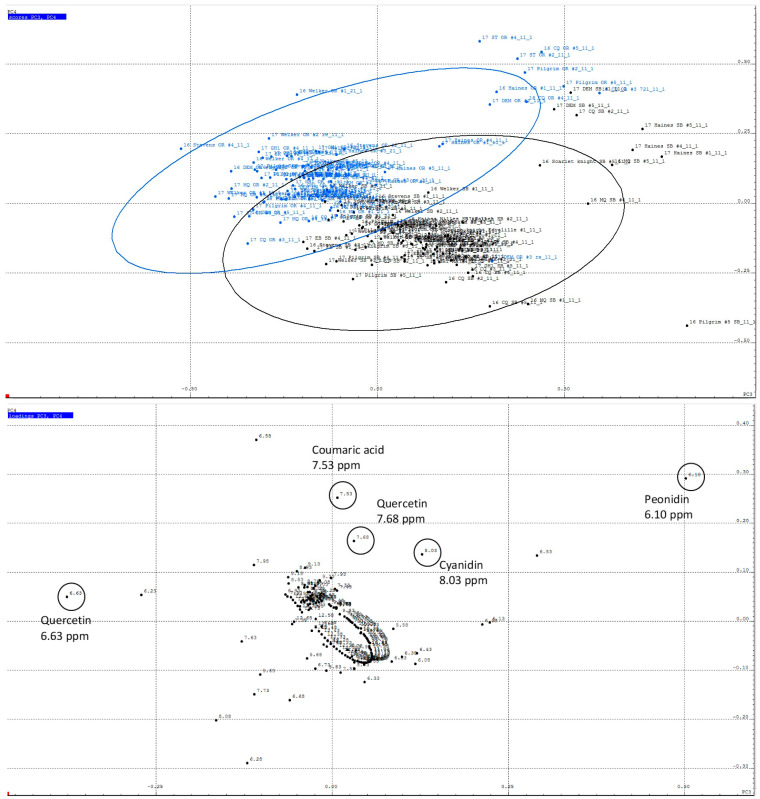
Principal component analysis (PCA) of ^1^H NOESY NMR spectra in the aromatic chemical shift range of 5.5–13.5 ppm. (**Top**) Scores plot showing a comparison of all samples by growing region (PC3 vs. PC4). Black dots represent MA samples; blue dots represent OR samples. (**Bottom**) Loadings plot showing contributions of each chemical shift bucket (black dots) to the regional variance and identification of selected metabolites. Chemical shift buckets corresponding to the labeled metabolites are circled.

PCA of ^1^H NMR data in the chemical shift range of 5.5 ppm to 13 ppm (Figure 3) indicates that some sample variation between regions can also be attributed to polyphenols. Some clustering of the MA and OR samples was evident along PC3 (6.77%) and PC4 (3.40% of variance), with OR samples tending towards higher PC4 scores and MA samples skewing lower. From the loadings plot, quercetin, cyanidin, peonidin, and coumaric acid could be identified as metabolites associated with chemical shift buckets contributing to the variance in NMR spectra between MA and OR samples. The contribution of the flavonoids to regional variation was subsequently confirmed by quantitative analysis of anthocyanins and flavonols using HPLC-DAD, discussed below.

### 2.2. Triterpenoids and Organic Acids Content

Quantitative proton ^1^H NMR (qNMR) methods were developed as a means to rapidly quantify triterpenoids and compare their content in cranberry fruit samples and supplements [26]. Reverse phase HPLC with UV or diode array detection is commonly used to detect phenolic acids and other flavonoids, including (+)-catechin, p-coumaric acid, myricetin, and quercetin, in cranberry samples through carefully designed separations [28,29]. However, it can be difficult to detect compounds lacking a chromophore (such as organic acids and triterpenoids) or to separate and identify isomers. Ursolic acid and oleanolic acid are isomeric triterpenes, differing only in the position of a single methyl group, making them harder to detect and distinguish through absorbance or LC-based quantification methods.

Quantitative proton ^1^H NMR (qNMR) has the advantages of universal detection of compounds without the need for chromatographic separation, simple, rapid analysis, and the ability to differentiate nearly identical compounds [30]. Hicks et al. quantified chlorogenic acid and hyperoside from crude blueberry extract with a LOQ of 0.01 mM using qNMR analysis [31]. QNMR has also been applied to the quantification of triterpenoids in Ganoderma resinaceum mushrooms used medicinally in China and Nigeria [32]. For our samples, qNMR worked best for analytes present at concentrations over 0.03 mM.

**Figure 4:**
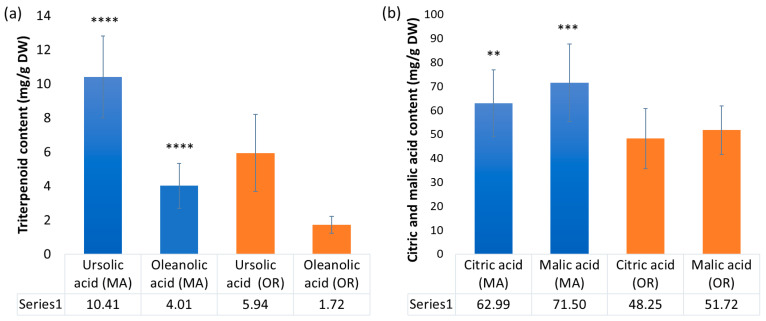
Regional comparison of (**a**) triterpenoids ursolic acid and oleanolic acid and (**b**) citric and malic acid in cranberry fruit, average of all cultivars over two growing seasons for MA vs. OR fruit (** = *p* ≤ 0.01, *** = *p* ≤ 0.001, **** = *p* ≤ 0.0001).

PCA identified ursolic and oleanolic acids as contributors to the regional variation in the secondary metabolite composition of the fruit. As these triterpenoids are also of interest from a health perspective, quantification was performed. Peak signals of selected metabolites were determined by comparison with previously reported NMR data by Turbitt and coworkers [26]. Table 1 shows the content of ursolic acid and oleanolic acid found in cranberry samples (average of five replicates) from each cultivar and region. Figure 1 shows relationships between common cultivars [27,33]. Ursolic acid was the predominant triterpenoid in all cranberry cultivars, with content ranging from 2.72 to 16.80 mg/g DW, followed by oleanolic acid, with content ranging from 1.07 to 7.89 mg/g DW. The cultivars Welker (MA) and Demoranville (MA) had the highest ursolic acid and oleanolic acid, respectively. Pilgrim fruit (OR) contained the lowest oleanolic acid. Triterpenoid content depended significantly on growing regions (Figure 4).

The average content of triterpenoids over both the 2016 and 2017 seasons across all cultivars from MA was significantly higher than the average across OR cultivars. Ursolic acid averaged 10.4 mg + 2.4 per g dry weight of fruit in MA samples and 5.94 + 2.27 mg/g dry weight in OR fruit. Likewise, oleanolic acid averaged 4.01 + 1.3 mg/g dry weight in MA fruit and 1.72 + 0.49 mg/g dry weight in OR fruit. Our previous study reporting on Early Black, Howes, and mixed cultivar fruit grown in MA found between 0.460 and 1.09 g of ursolic acid per kg of fresh weight of cranberry fruit, roughly equivalent to 5.0–11 mg/g dry weight [16]. A 2022 study of Red Star cultivar fruit grown in Lithuania reported 5.74 mg UA/g dry weight and 1.60 mg OA/g dry weight, comparable to the average quantity in our Oregon samples [34]. Somewhat lower quantities of UA and OA were reported for several cultivars grown at a cranberry plantation in Latvia [35]. A report on the 2016 cranberry fruit of several cultivars grown in Poland found ursolic acid at 1.0–1.76 mg/g dry matter and oleanolic acid from 0.89 to 1.14 mg/g of dry matter [36]. These triterpenoid levels were several times lower than what we detected in North American fruit, a difference which could be attributed to climate or other environmental factors. There is precedent for regional variation in the triterpenoid content of other fruit; a 2019 study of two apple cultivars grown across four locations in northeastern Europe, from Poland to Estonia, reported a significant regional influence on the content of ursolic and oleanolic acid in the fruits, with concentrations increasing from south to north [37]. The present study on East and West Coast cranberry fruit is the first report suggesting regional variation in content of two major triterpenoids associated with the anti-inflammatory and anti-cancer properties of cranberry fruit. This variation may be associated with climate, pest pressure, or other differences among the regions; further study is needed to elucidate specific contributing factors.

Using PCA, citric and malic acid were found to contribute to regional variation in phytochemical content, and this was confirmed by qNMR using methods we developed for cranberry supplements. Content ranged from 38.94 to 104.78 mg/g DW and 25.95 to 84.59 mg/g DW, respectively (Table 1). SK and GH1 cultivars averaged the highest citric acid; the HA cultivar was highest in malic acid, followed by CQ and WK. Citric acid was significantly higher in the MA samples overall (Figure 4), averaging 63 + 14 mg/g DW for Massachusetts (all cultivars) vs. 48.3 + 12.6 mg/g DW for Oregon cranberry fruit. Malic acid was also higher in MA fruit (71.5 + 16 mg/g DW) vs. OR fruit (51.7 + 10 mg/g DW).

### 2.3. Content of Anthocyanins and Flavonols

**Figure 5:**
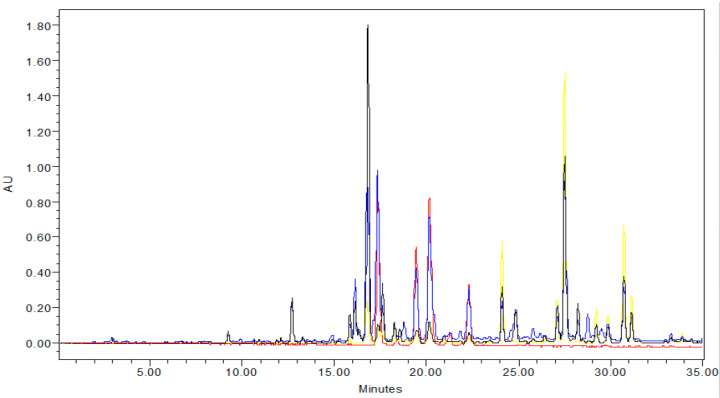
Representative HPLC-DAD chromatogram of ethanolic extract (ST MA 2016) showing the presence of anthocyanins (red, 520 nm), flavonols (yellow, 355 nm), and other phenolic constituents (blue, 280 nm, and black, 310 nm) detected at characteristic absorbance wavelengths.

Cranberries are a rich source of anthocyanins and flavonol glycosides. PCA of ^1^H NMR data in the chemical shift range of 5.5 ppm to 13 ppm indicated that some of the region-to-region sample variation can be attributed to quercetin glycosides, cyanidins, and peonidins. We have found ^1^H NMR less suitable for quantitative determination of cranberry flavonoids due to lower sensitivity and overlapping signals preventing reliable quantification [26]. HPLC-DAD analysis was instead employed to determine the content of individual anthocyanins and flavonols in the cranberry samples. Five predominant anthocyanin peaks were identified by HPLC-DAD (monitoring at 520 nm) eluting between 16 and 23 min; these were identified by matching retention time and UV absorbance patterns with those of authentic commercial standards and previously published data [38]: cyanidin-3-galactoside, cyanidin-3-glucoside, cyanidin-3-arabinoside, peonidin-3-galactoside, and peonidin-3-arabinoside. A representative HPLC chromatogram is shown (Figure 5). Cyanidins were quantified based on a cyanidin-3-galactoside standard, and peonidins were quantified based on a peonidin-3-galactoside standard (Table 2). Peonidin-3-galactoside was the most abundant anthocyanin. Peonidins in all fruit samples were higher than cyanidins. Total anthocyanins varied between 0.05 and 14.53 mg/g DW. Total monomeric anthocyanins in the fruit of multiple varieties harvested in New Jersey reportedly ranged from 12 to 86 mg/100 g of fresh fruit; when approximated for dry weight, this range is consistent with most of the cultivars analyzed in this study [20].

Seven flavonol glycosides were identified as eluting between 23 and 32 min (monitoring at 355 nm) by matching retention time and absorbance patterns with those of authentic commercial standards and previously published data [28,39]: myricetin-3-galactoside, myricetin-3-arabinoside, quercetin-3-galactoside, quercetin-3-xyloside, quercetin-3-arabinopyranoside, quercetin-3-arabinofuranoside, and quercetin-3-rhamnopyranoside. Total flavonol content was determined based on a quercetin-3-galactoside standard (Table 2). The most abundant flavonol was hyperoside (1.10–7.62 mg/g DW). Total flavonol content across all treatments ranged from 4.29–23.30 mg/g DW. Differences in anthocyanin and flavonol content were evident between cultivars, but there was no clear trend across all treatments. The Scarlet Knight cultivar was consistently the highest in total anthocyanins and flavonols for 2016 in both regions and among the highest in 2017. A UPLC-HRMS metabolomic study on fruit grown in New Jersey during 2017 found anthocyanins to be a strong contributor to cross-cultivar variations [27]. In the present study, metabolomic analysis of NMR spectra did not reveal significant differences in flavonoid content or clear groupings among the cultivars studied, but it did indicate significant variation in anthocyanin and flavonol content between growing regions.

**Figure 6:**
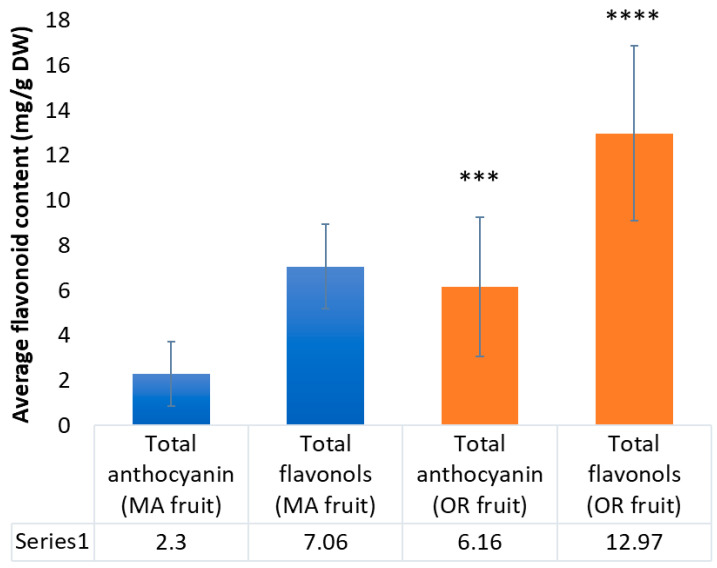
Regional comparison of the average total anthocyanins and flavonols for all cultivars over two growing seasons, OR vs. MA (*** = *p* ≤ 0.001, **** = *p* ≤ 0.0001).

In contrast with the triterpenoids and organic acids, the content of anthocyanins and flavonols, on average, was significantly higher in OR fruit than MA fruit (Figure 6). Total anthocyanin content across all OR cultivars (6.2 + 3.1 mg/g DW) was more than double that of the fruit harvested in MA (2.3 + 1.4 mg/g DW). Likewise, total flavonol content was much higher in OR fruit (13.0 + 3.9 mg/g DW) than MA fruit (7.1 + 1.9 mg/g DW). The trend in anthocyanin content can likely be explained by the influence of the environment on horticultural practices. Due to differences in climate and pest pressure, cranberries can remain in the bog longer after ripening without significant rot in coastal OR than in MA, allowing fruit to be harvested in OR into November while fruit in MA is typically harvested by early October. Season-to-season differences in the weather patterns may also impact phytochemical content; thus, we collected data on fruit from two growing seasons. MA experienced above-average temperatures and rainfall both years [40]. OR had average temperatures and rainfall during most of 2016 and 2017, but also experienced some of its highest recorded temperatures and very low humidity during August 2017 [41]. The effects of short-term weather extremes on cranberry phytochemical development are unknown at this time.

### 2.4. Proanthocyanidin Content

**Figure 7:**
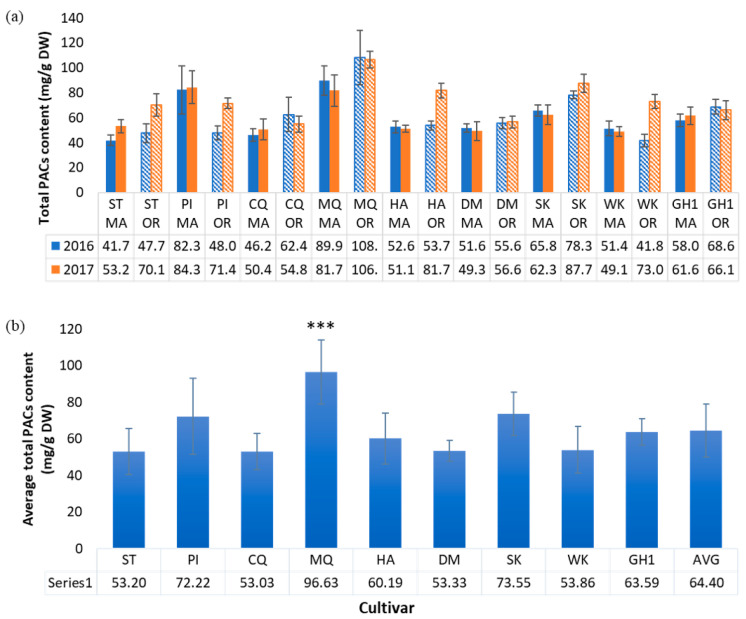
(**a**) Total proanthocyanidin (PAC) content by cultivar, growing region, and season, n = 5; (**b**) Average PAC content for each cultivar across all treatments, n = 20 (mg/g cranberry dry weight), *** = *p* ≤ 0.001.

The total PAC content of cranberry fruit was determined by the BL-DMAC assay using a cranberry PAC standard. Samples ranged between 41.7 and 108 mg PAC/g cranberry dry weight. Cultivar-based differences in PACs were observed (Figure 7), consistent with previous reports [20], but the averages across all cultivars did not differ significantly between the two regions. MQ exhibited the highest average PAC content (97 + 17 mg/g DW) across regions among the nine cultivars, significantly higher than the average, followed by SK (74 + 12 mg/g DW) and PI (72 + 21 mg/g DW). ST, CQ, and DM cultivars had the lowest average PAC content, around 53 mg/g DW. Across all cultivars, the average total PACs were nearly equal for 2016 OR (62.7 + 20.5 mg/g DW) and MA (60.0 + 16.4 mg/g DW) fruit. A higher average PAC content for 2017 OR (74.3 + 16.1 mg/g DW) vs. MA (60.4 + 13.8 mg/g DW) fruit was not statistically significant. The genetic background of various cultivars has been observed to influence PAC content in cranberries. Previous work by Rutgers University researchers determined the concentration of total PACs in eight cranberry cultivars grown in New Jersey using LC-MS [20]. The study found that Howes and its offspring #35 (Figure 1) exhibited the highest level of PACs. MQ, an offspring of #35, was also high in PACs. A 2014 study by Carpenter and coworkers examining proanthocyanidin content and composition in cranberry fruit of various cultivars grown in MA, NJ, WI, and British Columbia also reported cultivar-related variation in PACs. Some regional variation in PAC content was noted for MQ fruit, whereas CQ fruit had similar PAC content across three regions [42]. In the present study, MQ was highest in PACs among the nine cultivars, consistent with its genetic lineage (derived from Howes and #35) and with previous study data. We found CQ and DM among the lowest for PAC content, which is also consistent with Rutgers data. ST is the parent of CQ, which could explain its low PAC concentration. Another Rutgers study correlated fruit size with PAC concentration for ST and Ben Lear cranberries, showing an inverse correlation between PAC content and fruit size [39]. CQ and DM berries were the largest among MA fruit samples, and their PAC content was among the lowest. Cultivar appears to play a more significant role in determining PAC content, as demonstrated by the present study and others [20,27].

### 2.5. Trends in Total Phenolic Content

**Figure 8:**
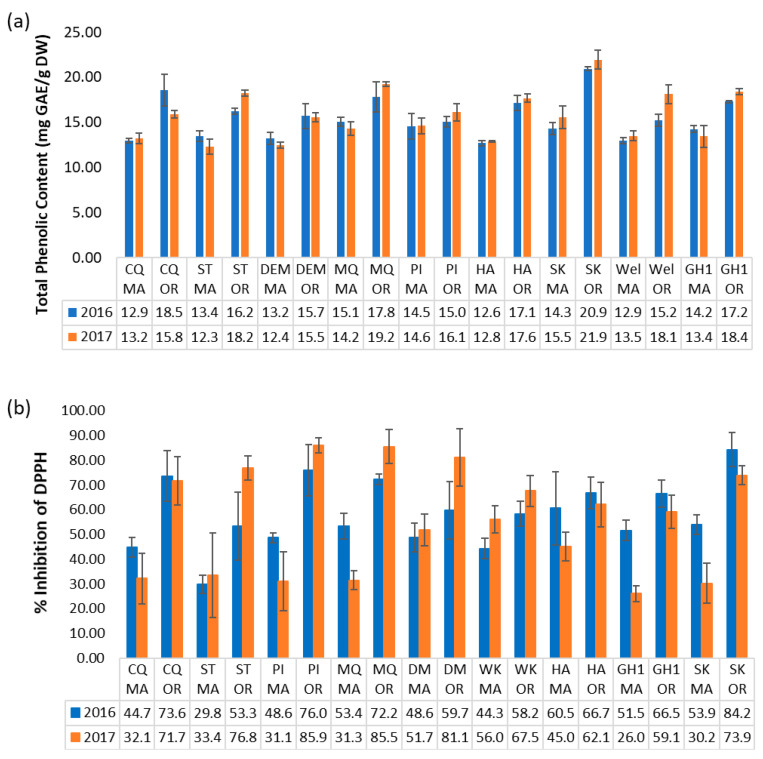
(**a**) Total phenolic content and (**b**) percent inhibition of DPPH radical by cranberry fruit extracts by cultivar, growing region, and season (n = 5).

**Figure 9:**
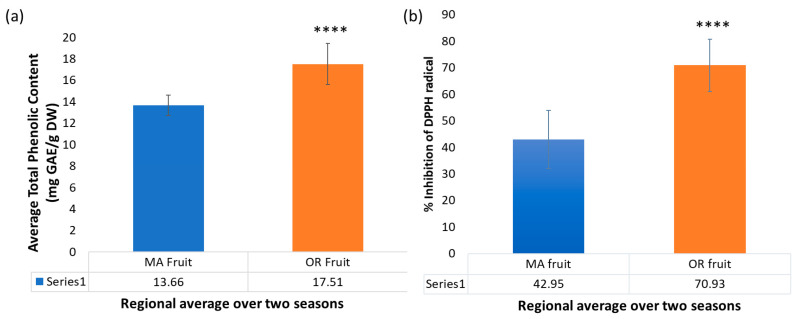
Regional comparison across all cultivars for (**a**) total phenolic content and (**b**) % inhibition of DPPH radical scavenging (**** = *p* ≤ 0.0001).

The Folin–Ciocalteu assay was used to estimate the total content of soluble phenolic compounds, or TPC (Figure 8a). This was significantly higher on average for OR fruit (17.51 + 1.91 mg GAE/g DW) than for MA fruit (13.66 + 0.94 mg GAE/g DW). OR fruit ranged from 15.07 + 0.57 mg GAE/g DW for PI cultivar to 20.94 + 0.19 mg GAE/g DW for SK cultivar in 2016, and from 15.55 + 0.53 mg GAE/g DW for DM to 21.95 + 1.01 mg GAE/g DW for SK in 2017. By comparison, 2016 MA fruit ranged from 12.69 + 0.29 mg GAE/g DW for HA to 15.09 + 0.49 mg GAE/g DW for MQ, and 2017 fruit ranged from 12.32 + 0.84 mg GAE/g DW for the ST cultivar to 15.58 + 1.27 mg GAE/g DW for SK. The averages across cultivars for OR vs. MA fruit are shown in Figure 9a. Scarlet Knight fruit from OR exhibited the highest TPC for both growing seasons, whereas Haines and Stevens fruit from MA had the lowest TPC in 2016 and 2017, respectively. The regional trend in TPC is consistent with the regional trend in the content of anthocyanins and flavonols. Cultivar-related variations in TPC have been previously reported for cranberries grown in Poland [43,44] or New Jersey [45]. Wang and Stretch [45] also reported an increase in TPC over three months in storage at temperatures above zero °C. Taken together, these studies demonstrate that phenolic content can depend on many factors, including genetic background, regional environment, cultivation conditions, maturation, and storage. The effect of pedigree on TPC was demonstrated in our study by DM and its offspring WK, both having similar TPC in 2016 MA fruit, and by CQ and its offspring HA for both growing seasons in MA (Table 2). SK was consistently among the highest cultivars for TPC. We also analyzed samples of Early Black fruit from 2017, a cultivar traditionally grown in MA. Extensive data on Early Black is not included in this study as the cultivar was not available at our OR site. Notably, Early Black fruit had the highest TPC (16.22 + 0.34 mg GAE/g DW) among all MA cultivars in our study, consistent with the results of Wang and Stretch [45].

### 2.6. Correlation between Antioxidant Activity and Metabolite Content

Cranberries are rich in flavonoids and other phenolics, which are likely to contribute to their antioxidant activity. The antioxidant capacities of cranberry crude extracts were determined by the DPPH free radical scavenging method. All samples were compared at the same extract concentration (250 μg/mL). In general, for both seasons and for most cultivars, extracts of the OR fruit samples inhibited free radical scavenging more effectively (53.32–85.93%) than their MA counterparts (26.05–60.56%) (Figure 8b). The average percent inhibition of DPPH radical by OR fruit extracts of all cultivars (70.8% + 9.9%) was significantly higher than the MA fruit (43% + 11%), as shown in Figure 9b. This was consistent with the regional trend in the content of anthocyanins, flavonols, and total phenolics and in contrast with the content of triterpenoids in cranberry fruit.

Linear correlation analyses were performed for antioxidant activity vs. key secondary metabolite groups (Figures S1–S6). Significant positive correlation was observed between antioxidant activity and the sum of total anthocyanins and flavonols content in all cranberry fruit samples during both the 2016 and 2017 growing seasons (R^2^ = 0.7189, *p* < 0.0001 and R^2^ = 0.7631, *p* < 0.0001, respectively) and over all treatments (R^2^ = 0.5043, *p* < 0.00001). This finding is consistent with positive correlations reported in a study of cranberry fruit grown in Poland [36]. In our study, SK and DM fruit samples from OR were among the highest in both antioxidant activity and anthocyanin content. Positive correlations were also observed between antioxidant activity and the total soluble phenolic content for the 2016 and 2017 growing seasons (R^2^ = 0.6351, *p* < 0.001; and R^2^ = 0.4364, *p* < 0.01, respectively) and over all treatments (R^2^ = 0.4808, *p* < 0.00001). Fruits with low total phenolic content demonstrated lower antioxidant activity, a trend also reported by Wang and Stretch [44]. However, no correlation was found between antioxidant activity and total PACs content either during the growing season (R^2^ = 0.1449 for 2016 and 0.0824 for 2017, respectively) or overall (R^2^ = 0.0868). This was surprising, as PACs are widely regarded as antioxidants; their contributions to radical scavenging may be secondary to those of other phenolics in the fruit.

In a related study of anti-inflammatory properties, we reported that concentrated extracts of the 2016 and 2017 SK and ST cranberry fruit from MA and OR significantly inhibited the LPS-induced expression of IL-6 in human THP-1 monocytes at 100 μg/mL [46]. Several of the extracts were inhibitory at 0.1–10 μg/mL. The data suggested that the observed anti-inflammatory activities were influenced by the content of flavonoids, total polyphenols, and triterpenoids. The data from the present study indicates a strong positive correlation between free-radical scavenging antioxidant activity and total anthocyanins and flavonols. In contrast, we observed that free-radical scavenging antioxidant activity in our samples demonstrated a slight negative correlation with the content of triterpenoids ursolic and oleanolic acid (R^2^ = 0.3513, *p* < 0.05 and R^2^ = 0.2707, *p* < 0.05 for 2016 and 2017 fruit, respectively; R^2^ = 0.224, *p* < 0.01 over all treatments). Reported antioxidant mechanisms for UA, OA, and related triterpenoids include upregulation of endogenous antioxidants such as catalase, superoxide dismutase, and glutathione, activation of the Nrf-2 pathway, and reduction of intracellular ROS production [15,47]. Further investigation of the antioxidant and anti-inflammatory mechanisms of cranberry triterpenoids is underway.

## 3. Materials and Methods

### 3.1. Plant Materials

Cranberry fruit from multiple cultivars was harvested from east coast Massachusetts (MA) and west coast Oregon (OR) cranberry bogs during the 2016 and 2017 growing seasons. Nine cranberry cultivars have been selected for this study, including Crimson Queen (CQ), Demoranville (DM), Mullica Queen (MQ), Pilgrim (PI), Welker (WK), Haines (HA), Grygleski Hybrid #1 (GH1), Scarlet Knight (SK), and Stevens (ST). Cranberry fruit from MA was harvested at the UMASS Cranberry Experiment Station in East Wareham, Massachusetts (41.76° N, 70.67° W) in late September and authenticated by Krystal DeMoranville. Cranberry fruit from OR was collected in late October from Bandon, OR (Coos County, 43.02° N, 124.43° W) and authenticated by Cassie Bouska (Oregon State University Extension Service, OR). Cranberry samples were selected randomly within each cultivar to yield five field replicates. All cranberry fruits were flash frozen with liquid nitrogen and stored at −20 °C until use.

### 3.2. Chemicals

4,4-dimethyl-4-silapentane-1-sulfonic acid (DSS) and deuterated dimethylsulfoxide (DMSO-d6) were obtained from Cambridge Isotope Laboratories, Inc. (Andover, MA, USA). Reagent-grade methanol and ethanol were purchased from Fisher Scientific (Hampton, NH, USA). Reagent-grade glacial acetic acid, hydrochloric acid, and acetone were purchased from Pharmco-AAPER (Brookfield, CT, USA), along with UV/HPLC-grade acetonitrile and methanol. HPLC-grade water was purchased from Honeywell (Morristown, NJ). Quercetin-3-galactoside (hyperoside) standard was purchased from ChromaDex, Inc. (Irvine, CA, USA). Standards of cyanidin-3-galactoside (assay ≥ 97%), and peonidin-3-galactoside (assay ≥ 95%) for HPLC analysis were obtained from Extrasynthese (Genay, France). Standards of ursolic acid (assay ≥ 90%), oleanolic acid (assay ≥ 97%) and 3,5-dinitrobenzoic acid were purchased from Sigma-Aldrich (St. Louis, MO, USA), along with N,N-dimethylaminocinnamaldehyde (DMAC) powder, 2, 2-diphenyl-1-picrylhydrazyl (DPPH), reagent-grade formic acid, gallic acid, and Folin–Ciocalteu reagent. Standards of benzoic acid and citric acid were obtained from J.T. Baker Chemical Co. (Phillipsburg, NJ, USA). 6-hydroxy-2,5,7,8-tetramethylchroman-2-carboxylic acid (Trolox) for the DPPH assay was purchased from Aldrich Chemical Company (Milwaukee, WI, USA). Cranberry proanthocyanidins (PAC) standard for the DMAC analysis was previously prepared from an isolated cranberry PAC fraction containing A-type flavan-3-ol oligomers [48], which was characterized by matrix-assisted laser desorption/ionization time-of-flight (MALDI-TOF) mass spectrometry.

### 3.3. Sample Extraction and Preparation

Whole cranberry fruits were partitioned into units of about 50 g (n = 5), freeze-dried via lyophilizer (LABCONCO FreeZone 4.5 L −84 °C), and ground to a fine powder (5~9 g). For quantitative analysis, cranberry powder from each replicate was split into sub-reps of 2.0 g and extracted in 100 mL of reagent-grade ethanol. Ethanol was chosen as a reliable universal solvent for most cranberry phytochemicals, including different classes of polyphenols and terpenoids. Solutions were stirred for an hour at room temperature and stored overnight at 0 °C. After vacuum filtration, the solid residue was re-extracted with 25 mL of ethanol, stirred for an hour at room temperature, then combined with the other extracts and rotary evaporated (Büchi Rotovapor R-200). Fruit extracts were freeze-dried to remove residual water and stored at −20 °C until use. These ethanol extracts were used for NMR, HPLC analysis, and DPPH assays.

### 3.4. Metabolomic Fingerprinting and Determination of Triterpenoids and Organic Acids Using Proton (^1^H) qNMR

A metabolomic fingerprint of each cranberry extract was acquired using ^1^H NOESY NMR, and a qNMR method was established to determine triterpenoids and organic acids in the whole cranberry extracts using the methods of Turbitt et al., 2020 [26]. Briefly, samples of each extract were prepared at 75 mg/mL in DMSO-d_6_ solvent. spiked with 0.1 mg/mL DSS as the internal chemical shift calibration standard, vortexed, and sonicated using a Fisher Scientific FS20H Sonicator (Pittsburgh, PA, USA) until fully dissolved. Samples were centrifuged, transferred to NMR tubes, and analyzed using a one-dimensional proton nuclear Overhauser effect spectroscopy experiment (1D ^1^H NOESY). NMR spectra were collected (ns = 8) at 298.0 K with gas flow at 400 lph on a Bruker AVANCE III 400 MHz NMR using Bruker TopSpin^TM^ 3.5. Metabolite identification, quantification, and chemometrics were performed with Bruker AssureNMR ^TM^ 2.0 and AMIX ^TM^ 3.9.15, using an internal NMR spectral database (SBASE, Bruker BioSpin, Billerica, MA, USA) as a reference. 3,5-dinitrobenzoic acid was chosen as an external calibration standard according to the PULCON approach [48]. Triterpenoids, including ursolic and oleanolic acids, were identified and quantified by methods described by Turbitt and coworkers [26], with automated matching of sample signals to well-characterized signals of purchased authentic standards in the SBASE. The calibration curves of these metabolites, including ursolic acid, and oleanolic acid produced suitable linearity, R^2^ = 0.9995 and 0.9998, respectively.

### 3.5. Determination of Flavonols and Anthocyanins by HPLC-DAD

High-Performance Liquid Chromatography with Diode-Array Detection (HPLC-DAD) was used to identify and quantify anthocyanins and flavonols within cranberry crude samples, according to previously published methods, with minor modifications [26]. Analysis was performed on a Waters Millennium binary HPLC system with two Waters 515 pumps at a flow rate of 0.9 mL/min, Millennium 32 version 32.0 software, coupled with a 996 photodiode array (PDA) detector. An Atlantis T3 column (4.6 × 150 mm, 3 μm; Waters) was used. Flavonols and anthocyanins were detected at 355 nm and 520 nm, respectively. The mobile phase consisted of 0.5% phosphoric acid/water as solvent A and water: acetonitrile: glacial acetic acid: phosphoric acid (50.0:48.5:1.0:0.5, *v*/*v*/*v*/*v*) as solvent B. Samples were dissolved in 25% methanol in solvent A and analyzed at room temperature with an injection volume of 20 μL. Anthocyanins and flavonols were identified by matching their HPLC peak retention time patterns and UV absorbance with those of authentic commercial standards and previously published data [26,28,38]. Quantification of cyanidins and peonidins was performed in comparison to external standard curves for cyanidin-3-galactoside and peonidin-3-galactoside, respectively; quantification of flavonol glycosides was based on an external standard curve for quercetin-3-galactoside (hyperoside).

### 3.6. Determination of Total Proanthocyanidin Content by DMAC Method

Total proanthocyanidins (PACs) content was determined using the BL-DMAC method described by Prior et al. with the cranberry PAC standard as previously described [49,50]. All samples of each replicate were tested in triplicate (5 replicates × 3, n = 15). Results are expressed as mg PAC per g DW.

### 3.7. Determination of Total Phenolic Content by Folin–Ciocalteu Assay

The total soluble phenolic content of freeze-dried cranberry samples was determined in triplicate using a microplate Folin–Ciocalteu method as described previously [51]. Briefly, 20 mg of each sample was sonicated in 95% ice-cold methanol (2 mL) for 30 min, then incubated for 24 h in the dark at room temperature. Samples were centrifuged at 13,000 g for five minutes. The supernatant (100 μL) was mixed with Folin–Ciocalteu reagent (10% *v*/*v*, 200 μL), briefly vortexed, then mixed with 700 mM aqueous Na_2_CO_3_ (800 μL) and incubated at room temperature for two hours. Samples (200 μL) were transferred to a 96-well microtiter plate, and the absorbance was read at 765 nm vs. a 95% methanol blank using a SpectraMax 190 microplate reader (Molecular Devices, San Jose, CA, USA). Total phenolic content was determined as gallic acid equivalents by comparison to a gallic acid standard curve.

### 3.8. Determination of Antioxidant Activity by DPPH Free-Radical Scavenging Assay

The free radical scavenging capacities of cranberry samples were determined by the 2,2-diphenyl-1-picrylhydrazyl (DPPH) free radical scavenging assay using a modified microplate method [52]. Cranberry ethanol extracts were dissolved in methanol to yield a concentration of 250 μg/mL, and then 200 μL aliquots were transferred to a 96-well plate (Costar, Corning, NY, USA). An amount of 50 μL of DPPH methanol solution (100 μg/mL) was added to each well, followed by a 30 min incubation in the dark. The final test sample concentration was 200 µg/mL, and the final DPPH concentration was 20 µg/mL. Absorbance was measured at 517 nm using a SpectraMax 190 microplate reader (Molecular Devices, San Jose, CA, USA). The blank was 250 μL of methanol, and the control contained 50 μL of DPPH methanol solution in 200 μL of methanol. Each field replicate was tested in triplicate (5 replicates × 3, n = 15). The free-radical scavenging antioxidant activity was expressed as percent inhibition using the following equation:

% inhibition = ((A_0_ − A_b_) − (A_1_ − A_b_))/(A_0_ − A_b_) × 100%

A_0_ is the absorbance of the control solution, A_b_ is the absorbance of the blank, and A_1_ is the absorbance of the remaining DPPH▪ in the presence of a cranberry sample.

### 3.9. Chemometric and Statistical Analyses

Non-targeted principal component analysis (PCA) of ^1^H NOESY NMR data was performed using AMIX ^TM^ 3.9.15 software, as previously described [26]. Briefly, spectra were partitioned into 0.05 ppm buckets, solvent regions were excluded, and variances were optimized via Pareto scaling. The principal contributors to variance between sample sets appearing in the Scores plots were identified by selecting the signal intensities that differ significantly between spectra and appear as outliers in the loadings’ plots. The confidence ellipsoids and minimum explained variance were set at 95% [25]. Metabolites contributing to the variance were identified by matching these signals to standards in the SBASE. Linear regression analysis was performed as conducted previously [46] using XLMiner to determine significant correlations between antioxidant activity and metabolite content. A one-way analysis of variance (ANOVA) was used to determine statistically significant differences between sample sets.

## 4. Conclusions

The present study illustrates a significant effect of the growing region in North America on the secondary metabolite composition and antioxidant properties of cranberries. PCA of ^1^H NOESY NMR data revealed specific metabolites (triterpenoids, organic acids, and flavonoids) that contributed to the variance between Oregon and Massachusetts fruit. Quantitation of these metabolites confirmed significant regional variation. Fruit from Oregon, a major growing region on the West Coast, exhibited significantly higher total flavonol and anthocyanin content over two harvest seasons than fruit from Massachusetts, a major growing region on the East Coast. By contrast, we found that MA fruit was consistently richer in triterpenoids and organic acids than fruit from OR. The influence of growing region on PAC content among fruit in our study was less evident, but PACs varied substantially among the cultivars. A strong positive correlation was observed for the antioxidant activity of cranberry fruits with the content of flavonols and anthocyanins, as well as with total soluble phenolic content, with OR fruit possessing greater overall anti-radical scavenging potential than fruit from MA. We report for the first time a regional influence (MA > OR) on the content of the triterpenoids ursolic and oleanolic acid, compounds that are likely to contribute to the anti-inflammatory benefits of cranberry fruit. Understanding patterns in cranberry metabolite production will contribute to improving harvest strategies and product development relating to the health-promoting properties of cranberry fruit. Cranberry flavonoids and triterpenoids have demonstrated potential for human health by protecting against oxidative stress and inflammation. The development of horticultural practices that maximize the content of these important compounds can provide greater dietary health benefits.

## Figures and Tables

**Table 1:** Content of triterpenoids and organic acids in cranberry fruit samples (mg analyte/g dry weight ± standard deviation of mean, n = 5).

Cultivar/Region	Growing Season	Ursolic Acid	Oleanolic Acid	Citric Acid	Malic Acid
ST/MA	2016	9.63 ± 2.39	4.23 ± 0.55	57.61 ± 9.00	43.78 ± 10.25
	2017	10.44 ± 3.28	3.83 ± 1.54	81.67 ± 10.91	54.98 ± 7.17
ST/OR	2016	7.09 ± 1.05	2.11 ± 0.68	38.69 ± 3.69	40.36 ± 8.91
	2017	7.01 ± 1.53	2.23 ± 1.18	47.86 ± 9.20	48.54 ± 7.73
MQ/MA	2016	9.02 ± 2.50	4.24 ± 1.18	50.89 ± 4.96	78.37 ± 12.07
	2017	9.61 ± 2.85	3.01 ± 0.90	67.31 ± 10.91	78.63 ± 9.16
MQ/OR	2016	6.25 ± 1.55	1.54 ± 0.28	47.81 ± 15.67	60.56 ± 7.72
	2017	12.32 ± 5.28	2.76 ± 1.58	34.97 ± 8.95	54.59 ± 8.29
DM/MA	2016	9.90 ± 2.57	3.59 ± 2.59	44.11 ± 9.49	75.68 ± 10.75
	2017	8.62 ± 2.66	4.27 ± 1.09	59.27 ± 12.83	79.92 ± 8.63
DM/OR	2016	3.18 ± 1.25	1.27 ± 0.06	45.86 ± 12.79	52.21 ± 6.24
	2017	6.68 ± 2.39	2.05 ± 0.36	53.85 ± 13.96	59.36 ± 5.81
PI/MA	2016	12.83 ± 1.78	5.80 ± 0.68	52.40 ± 9.48	51.43 ± 14.32
	2017	8.94 ± 2.35	2.49 ± 0.93	76.49 ± 18.91	66.00 ± 12.48
PI/OR	2016	4.31 ± 1.39	1.32 ± 0.99	52.41 ± 6.64	52.08 ± 8.70
	2017	3.09 ± 1.72	1.09 ± 0.34	63.30 ± 13.73	51.46 ± 7.87
CQ/MA	2016	9.18 ± 2.34	3.84 ± 1.22	64.30 ± 4.60	86.43 ± 10.69
	2017	9.50 ± 3.92	3.72 ± 1.70	84.59 ± 21.09	81.98 ± 10.72
CQ/OR	2016	2.72 ± 1.18	2.25 ± 0.65	25.95 ± 6.22	39.72 ± 4.46
	2017	5.60 ± 0.67	1.75 ± 0.60	58.71 ± 23.22	73.67 ± 12.36
HA/MA	2016	15.39 ± 4.31	7.60 ± 2.48	57.59 ± 8.39	88.27 ± 29.78
	2017	7.34 ± 1.20	2.60 ± 0.88	40.42 ± 15.04	104.78 ± 15.76
HA/OR	2016	5.92 ± 1.84	2.30 ± 0.81	64.37 ± 17.62	56.59 ± 12.12
	2017	6.10 ± 2.61	1.66 ± 0.89	30.97 ± 13.32	63.23 ± 8.55
SK/MA	2016	10.00 ± 2.27	3.39 ± 1.38	75.26 ± 16.41	58.19 ± 12.07
	2017	9.46 ± 3.41	3.25 ± 1.38	80.65 ± 14.47	57.94 ± 6.52
SK/OR	2016	6.01 ± 2.38	1.32 ± 0.60	61.52 ± 13.71	38.94 ± 6.82
	2017	8.20 ± 3.73	1.66 ± 0.57	51.80 ± 7.94	40.84 ± 6.99
WK/MA	2016	16.80 ± 9.95	5.99 ± 2.82	44.02 ± 10.32	76.37 ± 11.90
	2017	10.76 ± 2.87	3.05 ± 0.98	53.35 ± 2.87	88.29 ± 0.98
WK/OR	2016	4.40 ± 3.31	1.61 ± 1.11	34.46 ± 9.18	51.17 ± 9.33
	2017	4.72 ± 0.89	1.10 ± 0.27	33.50 ± 5.02	65.45 ± 11.28
GH1/MA	2016	11.46 ± 4.75	4.04 ± 1.38	68.13 ± 9.44	58.19 ± 1.38
	2017	8.45 ± 1.90	2.46 ± 1.16	75.84 ± 19.68	57.83 ± 9.26
GH1/OR	2016	5.16 ± 3.26	1.07 ± 0.32	65.68 ± 8.16	41.27 ± 3.94
	2017	8.20 ± 3.97	1.86 ± 0.82	56.71 ± 8.73	40.97 ± 5.21

**Table 2:** Content of flavonoids, PACs, and total phenolics in cranberry fruit samples (mg analyte/g dry weight ± standard deviation, n = 5).

Cultivar/Region	Year	Cyanidin Glycosides	Peonidin Glycosides	Total Anthocyanin	Quercetin-3-Galactoside	Total Flavonols	Total Phenolics ^a^	Total PACs
ST/MA	2016	0.87 ± 0.16	2.01 ± 0.38	2.88 ± 0.54	2.78 ± 0.67	6.88 ± 1.46	13.46 ± 0.59	41.71 ± 4.20
	2017	0.20 ± 0.13	0.42 ± 0.25	0.62 ± 0.24	2.58 ± 0.35	8.17 ± 1.07	12.32 ± 0.84	53.20 ± 5.15
ST/OR	2016	1.22 ± 0.28	4.29 ± 1.20	5.50 ± 1.48	3.77 ± 0.76	10.44 ± 2.94	16.25 ± 0.33	47.71 ± 7.67
	2017	0.55 ± 0.22	1.65 ± 0.67	2.20 ± 1.02	4.07 ± 0.90	13.37 ± 1.89	18.22 ± 0.35	70.17 ± 9.11
MQ/MA	2016	0.68 ± 0.11	1.57 ± 0.29	2.25 ± 0.39	3.75 ± 0.64	8.92 ± 1.65	15.10 ± 0.49	89.94 ± 11.68
	2017	0.09 ± 0.02	0.20 ± 0.03	0.29 ± 0.05	1.64 ± 0.37	5.42 ± 1.28	14.29 ± 0.74	81.77 ± 12.68
MQ/OR	2016	2.15 ± 0.59	7.13 ± 1.33	9.28 ± 1.90	5.00 ± 2.30	15.99 ± 5.71	17.83 ± 1.65	108.22 ± 22.1
	2017	1.05 ± 0.18	3.31 ± 0.30	4.36 ± 0.36	4.03 ± 0.77	13.01 ± 2.78	19.23 ± 0.24	106.58 ± 6.85
DM/MA	2016	0.79 ± 0.22	1.76 ± 0.50	2.55 ± 0.71	2.77 ± 0.65	7.86 ± 2.72	13.22 ± 0.69	51.69 ± 3.29
	2017	0.79 ± 0.42	1.59 ± 1.22	2.68 ± 1.61	1.95 ± 0.25	7.20 ± 0.79	12.48 ± 0.33	49.32 ± 9.54
DM/OR	2016	1.88 ± 0.89	7.33 ± 3.02	9.41 ± 3.90	4.11 ± 1.99	14.76 ± 7.38	15.72 ± 1.36	55.69 ± 4.37
	2017	1.39 ± 0.37	4.97 ± 1.44	6.36 ± 1.79	1.69 ± 0.29	7.12 ± 0.44	15.55 ± 0.53	56.64 ± 4.6
PI/MA	2016	0.95 ± 0.11	1.81 ± 0.17	2.75 ± 0.26	2.59 ± 0.51	7.04 ± 0.91	14.57 ± 1.39	82.39 ± 19.19
	2017	0.10 ± 0.03	0.16 ± 0.05	0.25 ± 0.06	2.09 ± 0.31	7.04 ± 0.66	14.64 ± 0.87	84.38 ± 13.16
PI/OR	2016	1.76 ± 0.52	4.20 ± 1.03	5.96 ± 1.56	4.54 ± 1.83	14.13 ± 5.44	15.07 ± 0.57	48.07 ± 5.59
	2017	0.94 ± 0.16	2.34 ± 0.30	3.28 ± 0.45	3.48 ± 0.39	12.15 ± 1.59	16.12 ± 0.96	71.40 ± 4.26
CQ/MA	2016	0.69 ± 0.14	2.03 ± 0.32	2.72 ± 0.46	3.16 ± 0.72	7.55 ± 1.78	12.95 ± 0.24	46.21 ± 4.93
	2017	0.52 ± 0.15	1.44 ± 0.59	1.97 ± 0.73	2.09 ± 0.58	7.63 ± 1.54	13.23 ± 0.62	50.49 ± 8.28
CQ/OR	2016	1.72 ± 0.36	5.76 ± 0.81	7.48 ± 0.86	6.62 ± 2.94	18.63 ± 5.84	18.58 ± 1.75	62.46 ± 13.68
	2017	0.85 ± 0.31	3.59 ± 1.82	4.44 ± 1.65	2.92 ± 1.25	10.11 ± 2.22	15.86 ± 0.42	54.84 ± 6.64
HA/MA	2016	1.15 ± 0.25	2.87 ± 0.40	4.02 ± 0.65	3.56 ± 0.82	10.19 ± 3.25	12.69 ± 0.29	52.63 ± 4.82
	2017	0.51 ± 0.09	1.33 ± 0.35	1.84 ± 0.40	1.10 ± 0.26	4.29 ± 0.79	12.89 ± 0.07	51.11 ± 2.97
HA/OR	2016	2.31 ± 0.10	6.76 ± 2.27	9.45 ± 3.11	6.00 ± 1.25	17.03 ± 3.33	17.15 ± 0.81	53.76 ± 3.78
	2017	0.55 ± 0.33	1.51 ± 0.95	2.05 ± 1.27	2.97 ± 0.65	10.55 ± 1.63	17.69 ± 0.47	81.77 ± 5.97
SK/MA	2016	1.81 ± 0.46	4.28 ± 1.08	6.10 ± 1.51	4.50 ± 0.96	11.37 ± 3.86	14.33 ± 0.67	65.86 ± 4.59
	2017	0.71 ± 0.39	1.53 ± 0.90	2.24 ± 1.28	1.77 ± 0.50	5.69 ± 1.78	15.58 ± 1.27	62.32 ± 7.82
SK/OR	2016	3.60 ± 0.72	10.93 ± 2.07	14.53 ± 2.72	7.62 ± 2.22	23.30 ± 8.25	20.94 ± 0.19	78.30 ± 3.03
	2017	2.03 ± 0.36	5.42 ± 0.98	7.45 ± 1.33	4.09 ± 0.74	11.72 ± 1.98	21.95 ± 1.01	87.71 ± 7.27
WK/MA	2016	0.88 ± 0.28	1.68 ± 0.42	2.56 ± 0.66	1.90 ± 0.52	5.65 ± 1.56	12.98 ± 0.31	51.48 ± 5.73
	2017	0.85 ± 0.11	2.71 ± 0.61	3.57 ± 0.67	1.68 ± 0.32	5.25 ± 1.63	13.51 ± 0.56	49.11 ± 3.83
WK/OR	2016	1.19 ± 0.38	4.37 ± 1.53	4.98 ± 1.69	2.13 ± 0.07	9.36 ± 1.57	15.22 ± 0.66	41.83 ± 5.14
	2017	0.85 ± 0.19	2.25 ± 0.62	3.11 ± 0.81	2.64 ± 0.91	10.18 ± 2.77	18.12 ± 1.05	73.02 ± 5.42
GH1/MA	2016	0.47 ± 0.13	0.95 ± 0.41	1.37 ± 0.57	2.31 ± 0.50	6.45 ± 1.25	14.27 ± 0.41	58.00 ± 5.07
	2017	0.24 ± 0.06	0.49 ± 0.15	0.73 ± 0.22	1.30 ± 0.52	4.41 ± 1.41	13.44 ± 1.24	61.66 ± 7.07
GH1/OR	2016	1.17 ± 0.20	4.26 ± 1.90	5.42 ± 2.05	3.26 ± 0.93	11.25 ± 1.90	17.29 ± 0.11	68.61 ± 5.92
	2017	1.33 ± 0.24	4.29 ± 0.92	5.62 ± 1.15	3.59 ± 0.82	10.35 ± 2.05	18.40 ± 0.34	66.10 ± 7.45

^a^ Total phenolics are expressed as gallic acid equivalents (GAE).

## Data Availability

Data are available in the article, Supplementary Materials, or by request to the corresponding authors.

## Supplementary Materials

The following supporting information can be downloaded at: https://www.mdpi.com/article/10.3390/plants12203595/s1, Figures S1–S6: Linear correlation data for antioxidant activity with total phenolic content, anthocyanins and flavonols, proanthocyanidins and triterpenoids.
