# Supplementary material for: Influence of the Growing Region on the Phytochemical Composition and Antioxidant Properties of North American Cranberry Fruit (Vaccinium macrocarpon Aiton)
Source: Plants (Basel). 2023 Oct 17;12(20):3595. doi: 10.3390/plants12203595 (PMC10609726; doi:10.3390/plants12203595)
Supplement: Supplementary file 1 [file plants-12-03595-s001.zip › plants-2655914-supplementary.pdf]

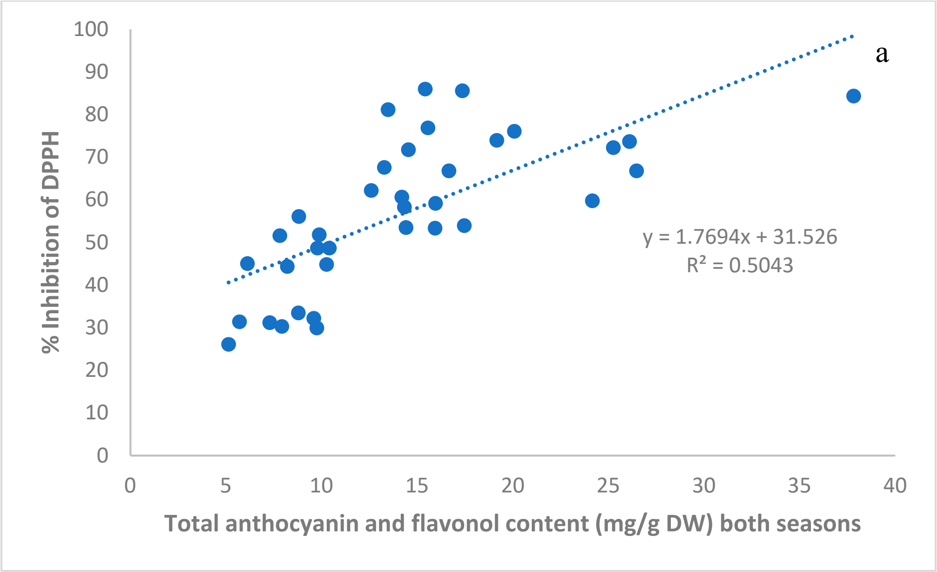

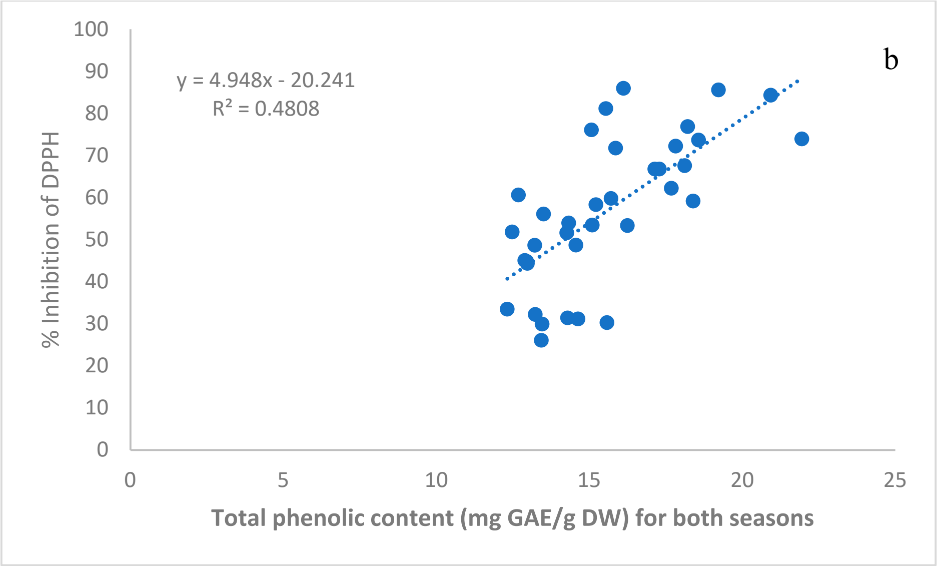

Figure S1: Linear correlation analysis data for all sample treatments, comparing antioxidant activity (% inhibition of DPPH radical) and (a) Total content of anthocyanins and flavonols, statistically significant positive correlation ($p = 0.0000021$); (b) Total phenolics content, statistically significant positive correlation ($p = 0.0000049$).

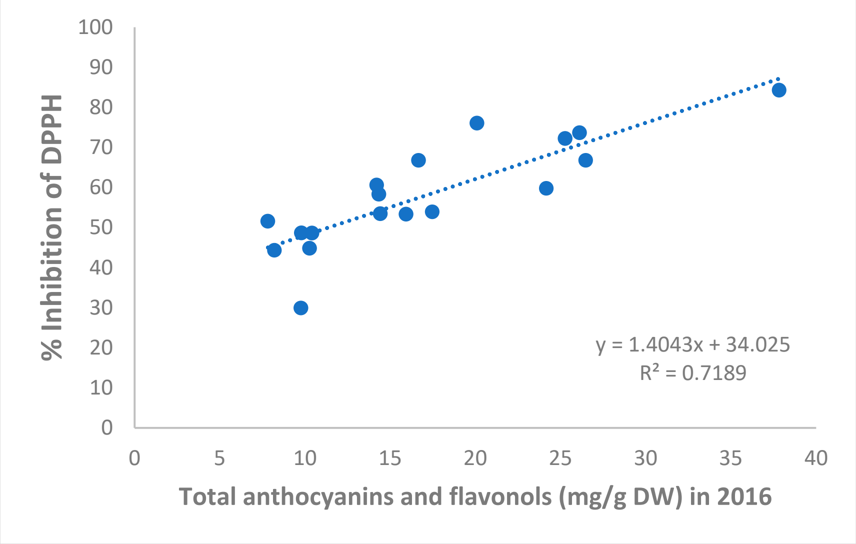
% Inhibition of DPPH
100
90
80
70
60
50
40
30
20
10
0
0
5
10
15
20
25
30
35
40
Total anthocyanins and flavonols (mg/g DW) in 2016
y = 1.4043x + 34.025
R² = 0.7189

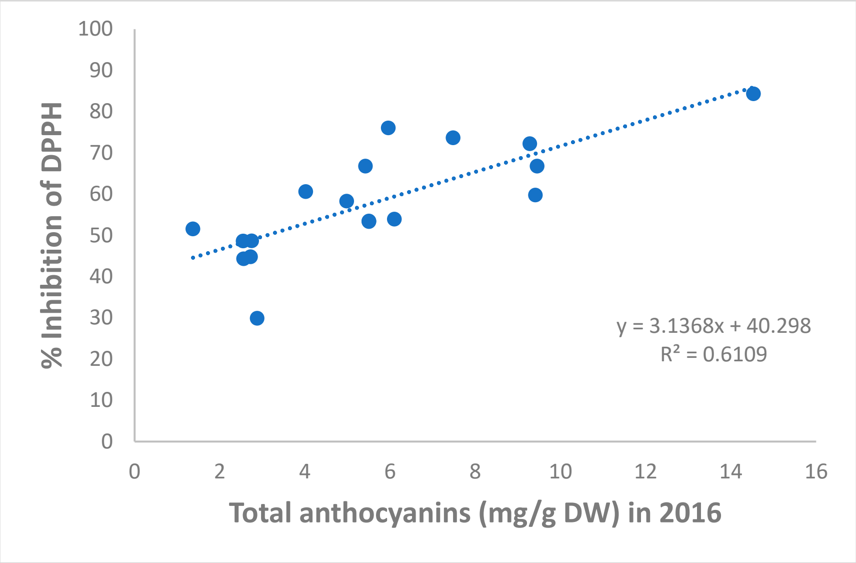
% Inhibition of DPPH
100
90
80
70
60
50
40
30
20
10
0
0
2
4
6
8
10
12
14
16
Total anthocyanins (mg/g DW) in 2016
y = 3.1368x + 40.298
R² = 0.6109

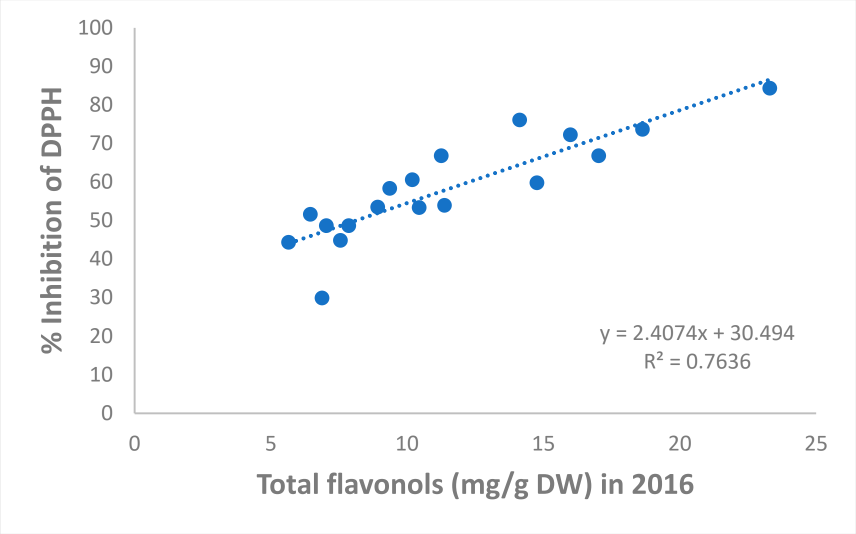
% Inhibition of DPPH
100
90
80
70
60
50
40
30
20
10
0
0
5
10
15
20
25
Total flavonols (mg/g DW) in 2016
y = 2.4074x + 30.494
R² = 0.7636

Figure S2: Linear correlation data for antioxidant activity with flavonoid content, 2016 fruit. Top: total content of anthocyanins and flavonols, significant positive correlation ($p = 0.000025$). Middle: total anthocyanins, significant positive correlation ($p = 0.00031$). Bottom: total flavonols, significant positive correlation ($p = 0.0000062$).

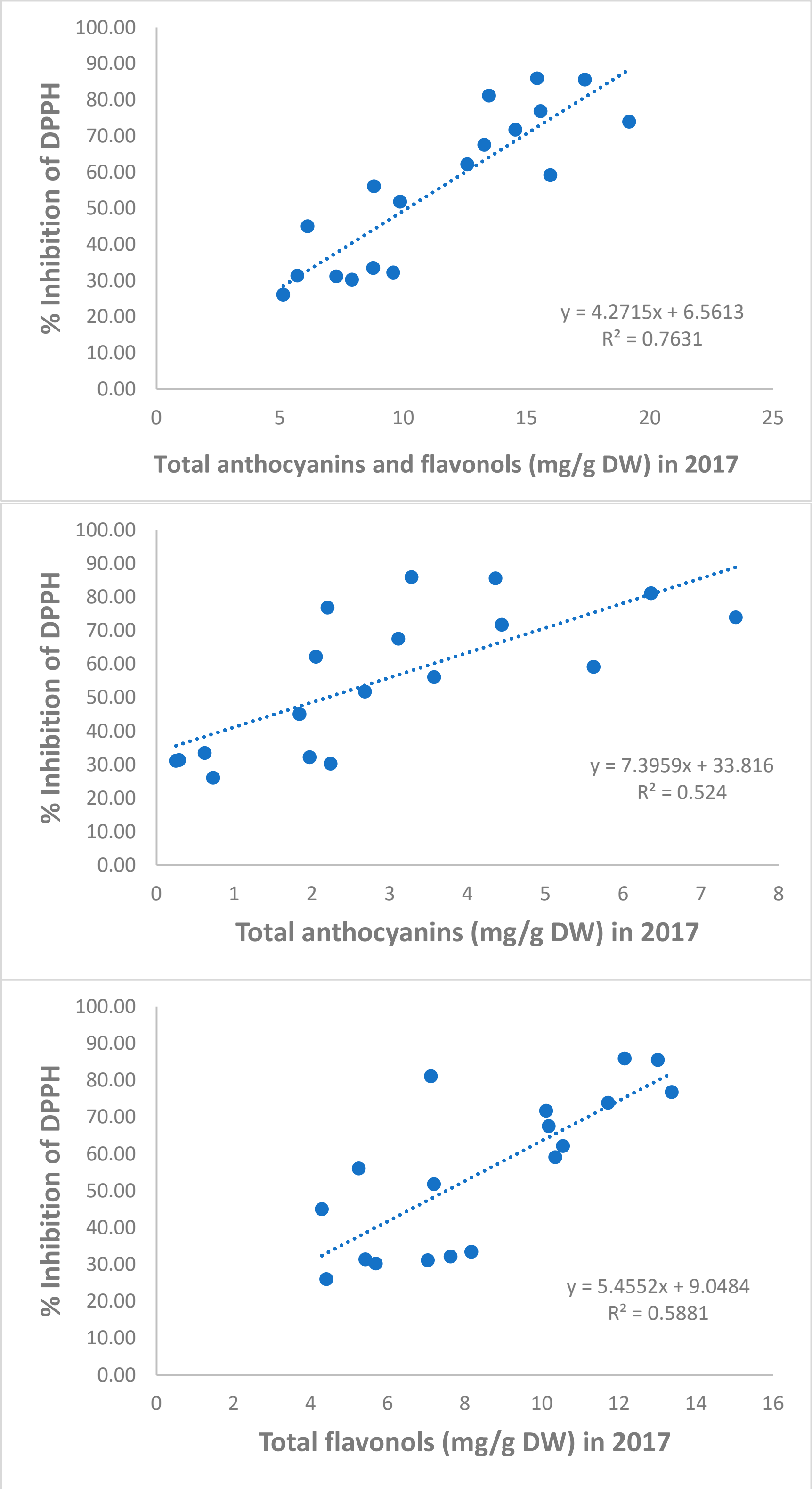

Figure S3: Linear correlation data for antioxidant activity with flavonoid content, 2017 fruit. Top: total content of anthocyanins and flavonols, significant positive correlation ($p = 0.0000027$).

Middle: total anthocyanins, significant positive correlation ($p = 0.001$). Bottom: total flavonols, significant positive correlation ($p = 0.00023$).

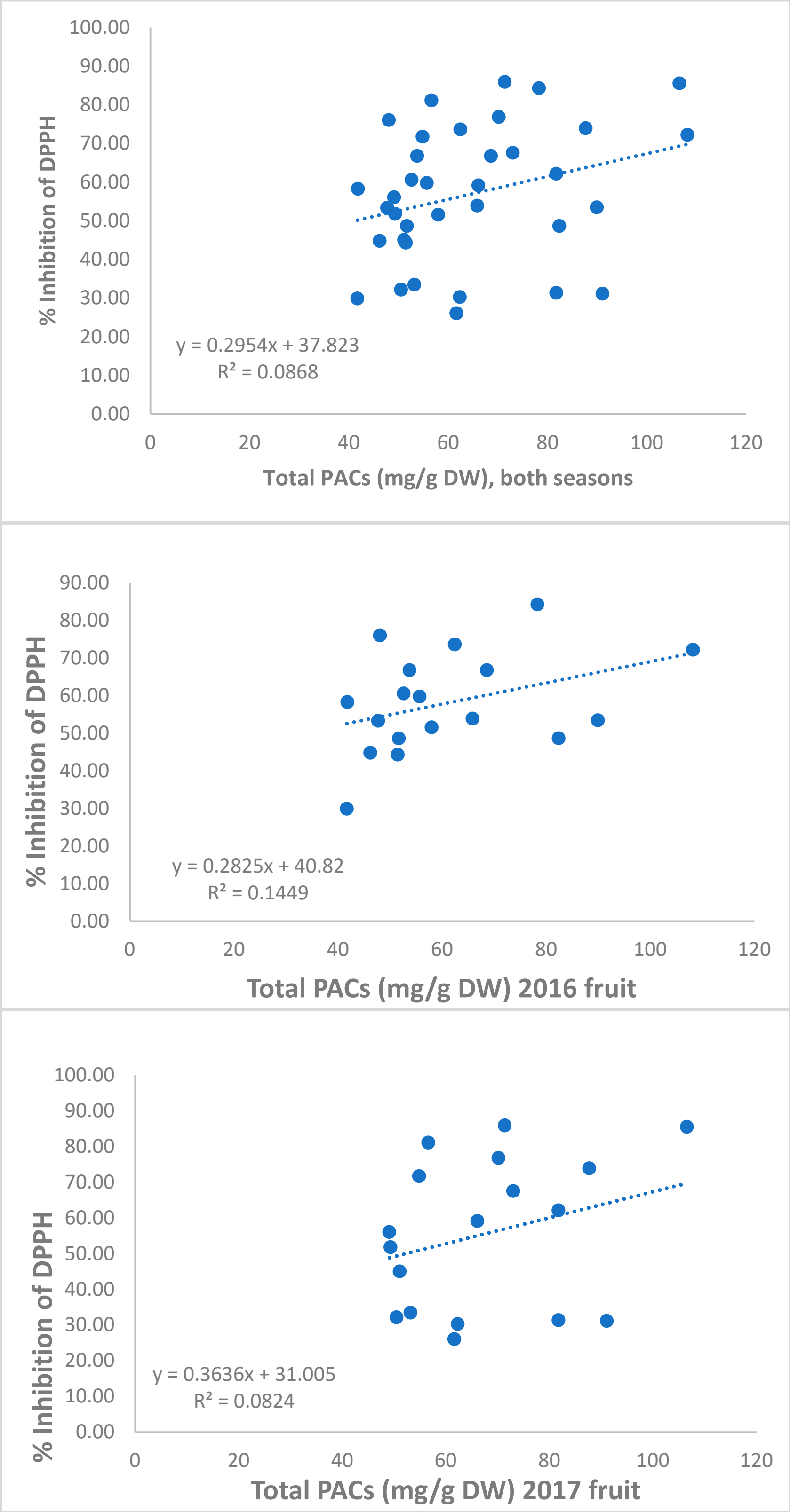

Figure S4: Linear correlation data for antioxidant activity with total PAC content. Top: All fruit treatments, no significant correlation (p = 0.322). Middle: 2016 fruit, no significant correlation (p = 0.173). Bottom: 2017 fruit, no significant correlation (p = 0.655).

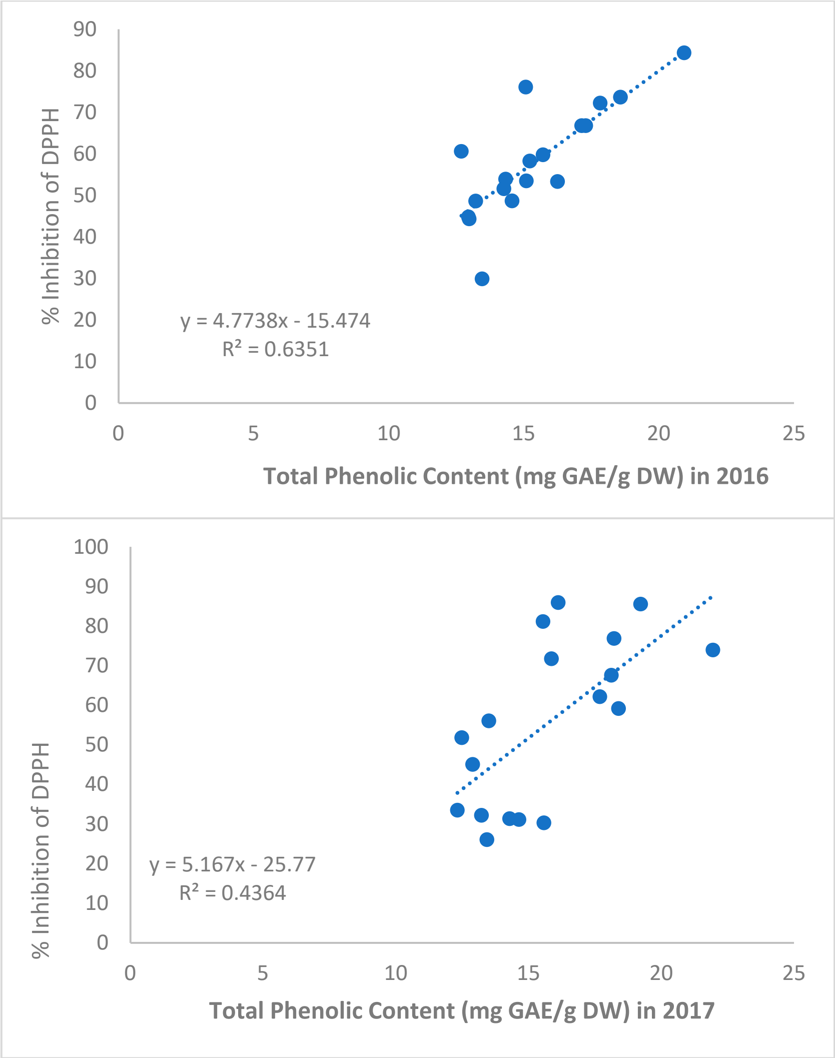

Figure S5: Linear correlation data for antioxidant activity with total phenolic content as measured by Folin-Ciocalteu assay. Top: 2016 fruit, significant positive correlation ($p = 0.000206$). Bottom: 2017 fruit, significant positive correlation ($p = 0.00587$).

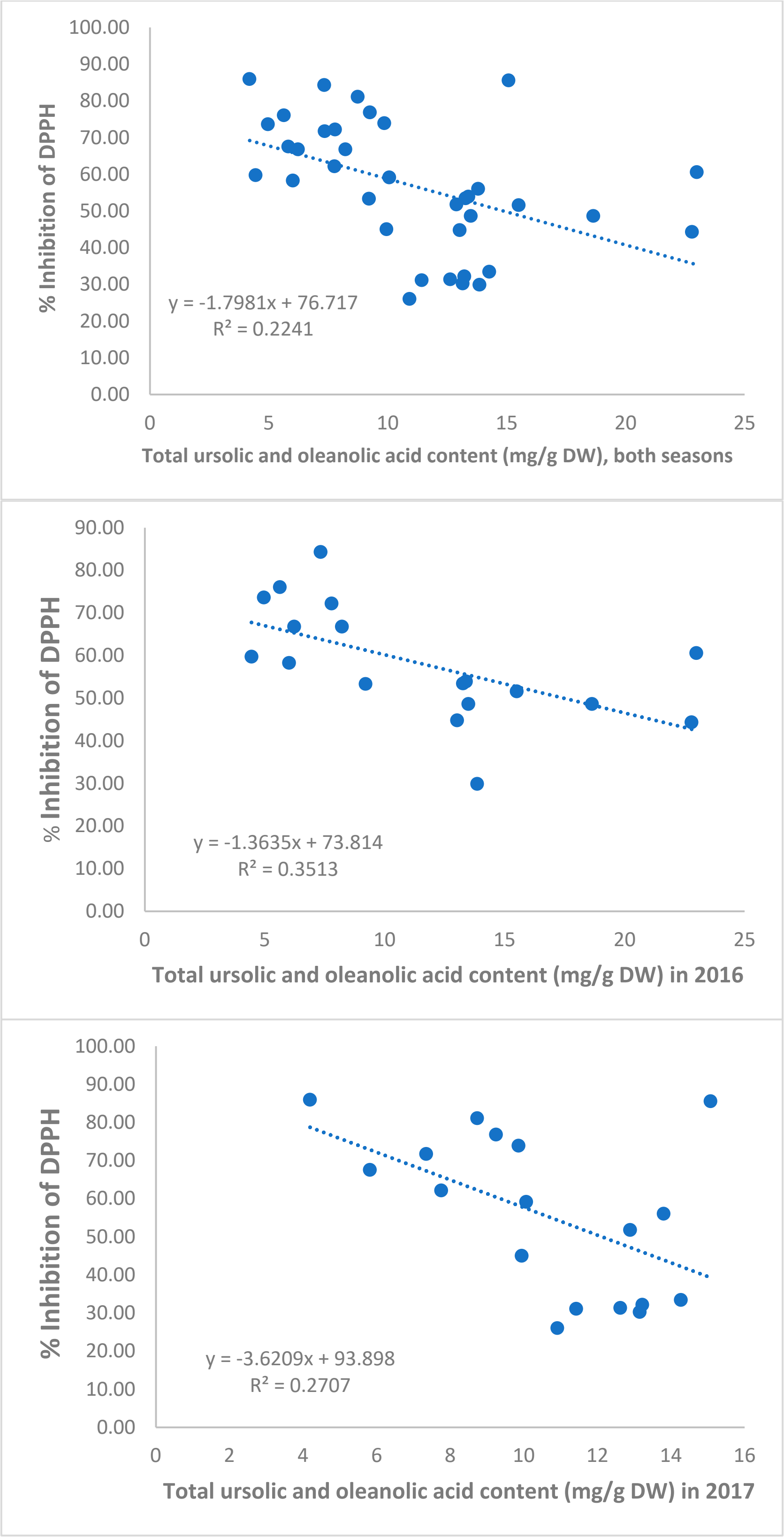

Figure S6: Linear correlation data for antioxidant activity with total content of triterpenoids ursolic and oleanolic acid. Top: All fruit treatments, significant negative correlation ($p = 0.00446$). Middle:

2016 fruit, significant negative correlation ($p = 0.0115$). Bottom: 2017 fruit, significant negative correlation ($p = 0.0456$).
